# The Comparison of Marital Satisfaction and Mental Health in Genital Mutilated Females and Non-Genital Mutilated Females

**DOI:** 10.5812/ijhrba.5365

**Published:** 2012-11-26

**Authors:** Anahita Khodabakhshi Koolaee, Taghi Pourebrahim, Bayan Mohammadmoradi, Mansor Ali Hameedy

**Affiliations:** 1 Department of Behavioral Sciences, University of Social Welfare and Rehabilitation Sciences, Tehran, IR Iran; 2 Department of Counseling, Faculty of Psychology and Educational Sciences, Shahid Beheshti University, Tehran, IR Iran; 3 Department of Counseling, Shahid Beheshti University, Tehran, IR Iran; 4 Department of Educational Psychology, Faculty of Psychology and Educational Sciences, Alzahra University, Tehran, IR Iran

**Keywords:** Female, Genital Mutilation, Couple Satisfaction, Mental Health

## Abstract

**Background:**

Female circumcision, sexual amputation, or Female Genital Mutilation (FGM) is an action existing in some cultures and regions of the world. For instance, in Africa and some little communities in Middle East use this operation for many reasons. This operation is forbidden in those countries and is never linked to religious attitudes.

**Objectives:**

The purpose of the following research was to determine and compare marital satisfaction, mental health and sexual satisfaction between Kurdish genital mutilated and non-genital mutilated females.

**Patients and Methods:**

To achieve the goal of research, a sample of 200 married females of Ornament area in Kermanshah province were selected by non-randomized sampling Enrich Marital Inventory, and General Health Questionnaire was used for data gathering. Data was analyzed between the two groups by utilizing independent t- test. The significant level was P < 0.05.

**Results:**

The findings indicated that there was a significant difference between genital mutilated females and non-genital mutilated females. Besides, the results revealed that two groups of participants had significant difference in mental health and the other subscales like physical symptom and sleep disorder from general health.

**Conclusions:**

It is noteworthy to point out that there is strong relation between sexual desire and marital satisfaction and their fulfillment reflects in mental health of people. So, when women experience the FGM in their lives, in fact they lose the gratification of their whole life. Awareness of the mental and psychical dangerous results of this operation could help women and prevent doing this inhuman action to others.

## 1. Background

Family is the most important institution of the society and it is the most significant unit of social behavior among human beings. Many researches in humanities point out the impact of this institution on forming the behavior of its members. Many of the mental health professionals and researchers improved that in person life satisfaction is related to fulfill most of the needs in family environment. When a couple decide to make their life the role of each spouse is very important. Couples do their efforts to create an atmosphere which can satisfy all their needs. The marital satisfaction consists of a sense of consent, experiencing consent and enjoyment by husband and wife, concerning all different aspects of life (1). But occasionally some problems could threaten the happiness of couples and consequently the whole family. Marital satisfaction plays a key role in fulfillment of couples’ needs and depends on some elements including personal issues, marital relationship, problem solving, financial management, leisure, sexual relationship, and relatives and friends. When the most important element of marital satisfaction like sexual satisfaction is endangered it could damage couples’ relationship or threaten their mental health. Many factors could affect the sexual function of people, but some of them are not genetic or stem from diseases, they are caused by some customs, and unfortunately root in their irrational and wrong Beliefs .Female Genital Mutilation (FGM) is one of them which has a long history and is still a common custom in some parts of the world. According to definition of the World Health Organization (WHO,2010), Female Genital Mutilation (FGM) are all the methods which lead to mutilation or cutting all or a part of female genital organism which are done on the basis of cultural goals or other unhealthy reasons (2). This action might be done at four different levels ranging from cutting and taking all or a part of clitoris to take out the large lips of external genital organism. The historical off spring of cutting female genital organism is not really known, but there are some records of doing it, in Egyptian mummy corpses. Clitoris is one of the sensitive tissues in female genital organism and its stimulation can lead to female orgasm. In female genital mutilation a part of female genital organism such as clitoris is cut, which results in decrease of sexual and marital satisfaction (3) clitoris and pre-derma are connected, in the case of girl genital mutilation sexual problems are caused in 75% of cases. Although pre-derma isn’t removed, the connection to clitoris is cut here. Despite adopting strategies and showing female anger and roars and fighting against FGM, five years after holding female conference in Beijing, both, Female genital mutilation and prostitution are the phenomena which haven’t had any concerned results yet. Some attempts have been made by specific organizations dependent on united nation organization, female community and defense over human rights and even from the countries that, this phenomenon is committed there. The world health organization states that 130 million women and girls have had genital mutilation in 25 countries of Africa and Middle East; not only it has not decreased, but also every year two million individuals are added to (4). According to the last statistics of the world health organization, almost more than 140 million females such as children, teenagers and youngsters are mutilated in more than 30 African and Asian countries. More than 79% of girls in African countries and 12% in Asian countries are victims of this issue (2). In some countries in West Africa and North Africa like Egypt and Sudan use this method to prevent the women to have sex before marriage. These attitudes rooted in historical and ancient thoughts, Islam and other religions have forbidden FGM. The majorities of FGM operations are illegal and are made by unprofessional and uneducated persons. Some of these operations have many physical and mental consequences, including damage of genital organs, serious infections, and sexual desire disorders (5). To decrease the harmful results of this inhuman action toward women and to provide awareness of risks and dangers of FGM, field researches were needed. People in “Kang Harbor” believe that there are evil impacts in Genital organ of women which can be kept away only by genital mutilation. Of course, it should be considered that this matter is quite logical in their culture because they look at it, as an ancient custom and the critics are called rebellious and guilty. Surly, it should be known that, the action is not just limited to “Kang”, and it is prevalent in some tribes of “Lorestan” and “Khuzestan” province, and it is justified in different ways. The female genital mutilation is prevalent among some ethnical and religious minorities which have justified it as a cultural and sanitary action. Mutilating a part of female genital organism is very painful and has a lot of physiological effects. Genital mutilation diminishes the sexual desire of female to the highest levels. Historically, this action was prevalent among Arabs, but nowadays it is common among some Arabic groups especially in North Africa and also, at the present time, in the west and south of Iran (5). Clitoris and pre-derma are connected and girl genital mutilation makes sexual problems in 75% of cases. Although pre-derma is not removed, the connection to clitoris is cut. According to UNICEF, female genital mutilation violates the basic rights of female and girls and damages their health (6). Alariqi performed a research in Yemen and concluded that 24% of female are circumcised or genital mutilated, dissatisfaction of sexual relationship in this group was more than female without genital mutilation (7). Khodabakhshi Koolaee (5) in a research under the title of report of Kurdish female genital mutilation found that 80% of female mutilated in their childhood have reported the sense of pain in genital region and dissatisfaction of sexual relationship with their husbands (5). Although the psychological aspects of female genital mutilation are not examined, the stress and horror before cutting genital organism is prevalent in the girls aware of this action. Regarding to several examinations done by World Health Organization (WHO), it is concluded that there is no medical and sanitary advantages in supporting the different levels of female genital mutilation (2). Most of Female Genital Mutilation (FGM) are operated by old women who are working as local physicians, they are uneducated and do not have any information about women genital organs and their tools of operation are unsterile and unhealthy. Some of the young girls operated under these conditions had infections in their genital organs. FGM could affect marital satisfaction and endanger the marital life. Also, when couples are not satisfied in the marital life, could not respond to the sexual needs and demands of their spouse and themselves, it is very clear that their mental health could affect their marital satisfaction. FGM women lose the key organ of their vagina and sexual satisfaction is affected by mutilation. The world health organization defines mental health as a complete healthy state; it means, a person should be healthy regarding physical, psychological and social items, and he shouldn’t have sickness and physical disability. We know that when a person understands his tendencies, provocations, dreams, and stimulations can be called as an individual with psychological health. Different researchers have shown that, life satisfaction anticipates mental health. Life satisfaction is different from other psychological constructs, such as positive and negative sentiment, self-respect and optimism (8). Having sexual satisfaction is one of the marital satisfaction items. The rate of couples’ satisfaction from sexual relationship and the ability to take and give pleasure to each other are called sexual satisfaction. The psychological effects of FGM have a deep influence on child’s mind and it can be a reason for behavioral disorders. The sense of uncertainty in childhood period could be leading the psychological harmful effect in further. After a while, females may suffer from the sense of deficiency, depression, chronic stimulation, frigidity, struggling with husband, reverse reflex and even psychos. Most of the women suffering from FGM, may not be able to express their feelings and horror and may live in isolation (9). Being aware of Female Genital Mutilation problems is quite necessary for people. Some people do not have enough knowledge and information about the harms of FGM in women and young girls. Researches are needed to clarify the mind of mental health professionals and also, the governors to enact the rules to prevent of this inhuman action. Although, in Iran and most of the countries this action is forbidden and government recognizes it as a criminal act, in far villages and some minority groups like Kurdish people FGM exists. Culture is a multi-componential matter. The culture of a society could be an impact of beliefs and ideas of people. But sometimes the beliefs made by irrational and wrong ideas dramatically endanger mental and physical health. Unfortunately, some irrational customs are still alive in villages and minority groups in Iran. One of the cultural issues in Oramanat area in Kermanshah Province is the issue of female genital mutilation. According to research goals mentioned above, these questions were examined. They were including:

Is there any difference between marital satisfaction of genital mutilated females and non-genital mutilated females, especially sexual satisfaction?

Is there any difference between mental health of genital mutilated females and non-genital mutilated females?

## 2. Objectives

The current research aimed to determine and compare marital satisfaction, mental health and sexual satisfaction between genital mutilated and non-genital mutilated females.

## 3. Patients and Methods 

Participants were 200 married females of Oramanat area of Kermanshah Province, 100 genital mutilated females and 100 non-genital mutilated females, who were selected by non-randomized sampling, completed ENRICH marital satisfaction questionnaire and 28-questions General Health Questionnaire (GHQ). ENRICH questionnaire: The questionnaire was designed by Olson, Fournier, and Druckman (10), in order to evaluate the potential problem-making issues or to identify enriching and nurturing relationship issues. The questionnaire is also used to identify the couples who want to enrich their relationships and need advice. Reliability: According to alpha coefficients of “Enrich Questionnaire”, reliability of the test in Olson, Fournier, and Druckman (1983) for sub measures of ideal distortion, marital satisfaction, characteristic subjects, relationship, solving problem, financial management, leisure, sexual relationship, children and growing child, the rules of demanding justice are regularly as follows: 0.71, 0.72, 0.77, 0.0, 48.76, 0.74, 0.75, 0.68, 0.73, 0.81, 0.9 respectively (10). Validity: Coefficient correlation of “enrich questionnaire” has the measures of family satisfaction from 41% to 60% and it has the measures of life satisfaction from 32% to 41%, as construct validity. All sub measures of “enrich questionnaire” which distinguish the satisfied and unsatisfied couples, have appropriate criterion validity. GHQ-28 questionnaire: The questionnaire was invented by Goldberg and Blackwell and planned to identify psychic disorders in different centers and environments (11). The questions examine the mental condition of individuals in a recent month, and they consist of signals such as, thoughts and normal emotions and some aspects of visible behavior. There are several forms of this instrument including the 60, 30, 28, and 12- item forms. The 28-item form of general health (GHQ-28) was utilized in this research; it measures four sub measures in psychopathology (11). Validity: Goldberg and Blackwell have reported rate of disorder in coefficient correlation among the questionnaire and the results of clinical evaluation as 80%. Chan and Chan have done a research by GHQ questionnaire, in order to examine general health of a group of 224 individuals in china. According to the method of construct measuring of the Likert spectrum, they reported that the stability and internal consistency equals to 0.85 by Cronbach’s alpha method (12). Hankins reported the reliability (13) for GHQ with Cronbach’s Alpha method as 0.90 (13). The study was cross- sectional. Two groups of participants were compared including two variables; marital satisfaction and General health questionnaire. Data was analyzed between the two groups by utilizing independent t- test for two groups. The significant level was determined (P < 0.05). SPSS versaion-16 was employed to analyze the data.

## 4. Results

In Table 1, the results of socio-demographic characteristics of all of participants are indicated. As shown in Table 1, the high category of age belonged to 25 - 30 and high level of education was for primary school participants. Also, 82 percent of participants were living in Urban and 18 percent of them were living in rural areas.

**Table 1.** Socio - Demographic Characteristic of all Participants

	No. (%)
**Age – Group, y**	** **
15-20	37(20)
21-25	63 (30)
26-30	72 (40)
31-35	28 (10)
**Educational level**	
Primary school	74 (37)
Secondary school	66 (33)
University or above	60 (30)
**Place of living**	
Urban	164 (82)
Rural	36 (18)

Regarding the results of Table 2, the t- test indicated that there was a significant difference among the items of personal issues, marital relationship, solving problem, financial management, leisure, sexual relationship and relatives and friends and total of two groups of participants. The comparison of average of scores between two groups indicated that FGM group had higher score in all sub-scales, except regional tendency than Non- FGM group. Regarding the results of Table 3, the t- test indicated that there were a significant difference between the two groups of participants in items of physical symptoms, sleep disorder, general mental health between genital mutilated females and non-genital mutilated females.

**Table 2.** Results From t - Test Analysis of Comparing the Means of Two Samples on Marital Satisfaction and Subscales ^a^

	Mean ± SD	*t*	*P*
**Personal subjects**		-4.701	0.001
FGM	14.12 ± 4.156	* *	* *
Non-FGM	16.70 ± 3.583	* *	* *
**Marital relationship **		-7.118	0.001
FGM	13.71 ± 4.075		
Non-FGM	4.050 ± 17.80		
**Solving problem**		-4.186	0.001
FGM	3.345 ± 14.98		
Non-FGM	2.735 ± 16.76		
**Financial management**		-2.96	0.003
FGM	4.546 ± 16.24		
Non-FGM	4.457 ± 18.13		
**Leisure**		1.968	0.05
FGM	4.553 ± 15.06		
Non-FGM	2.534 ± 15.76		
**Sexual relationship**		-2.38	0.001
FGM	2.474 ± 12.00		
Non-FGM	3.259 ± 21.69		
**Marriage of children **		-1.790	0.75
FGM	2.848 ± 17.00		
Non-FGM	3.275 ± 17.92		
**Relatives and children **		-4.814	0.001
FGM	3.961 ± 15.66		
Non-FGM	3.632 ± 17.91		
**Religious tendency **		-1.325	0.187
FGM	2.940 ± 18.18		
Non-FGM	3.911 ± 18.84		
**Total**		-8.63	0.001
FGM	3.436 ± 13.69		
Non-FGM	21.07 ± 15.68		

Abbreviation: FGM, female genital mutilation

^a^Each group consists of 100 subjects, df = 198

**Table 3.** Results From t - Test Analysis of Comparing the Means of Two Samples on Marital Satisfaction and Subscales a

	Mean ± SD	*t*	*P*
**Physical symptom**		2.18	0.045
FGM	7.100 ± 4.53	* *	* *
Non-FGM	5.860 ± 4.14	* *	* *
**Sleep disorder**		3.284	0.001
FGM	7.840 ± 4.70	* *	* *
Non-FGM	5.842 ± 3.86	* *	* *
**Dysfunction**		0.236	0.814
FGM	8.180 ± 3.84	* *	* *
Non-FGM	8.070 ± 2.64	* *	* *
**Depression**		1.541	0.125
FGM	4.680 ± 4.58	* *	* *
Non-FGM	3.750 ± 3.92	* *	* *
**Total**		2.94	0.23
FGM	27.80 ± 14.15	* *	* *
Non-FGM	23.52 ± 1172	* *	* *

Abbreviation: FGM, female genital mutilation

^a^Each group consists of 100 subjects, df = 198

Regarding to this reality that low grade showed the high mental health and considering the comparison of both groups’ averages, it was indicated that non-genital mutilated females had more mental health (Table 3).

## 5. Discussion

As shown in Table 1, 37% of participants belonged to 25-30 age category. Also, 40% of them had primary school education. In addition, 82% of participants were living in Urban and 18% of them were living in rural are as indicated in Table 2, there was a meaningful difference in rate of marital satisfaction between genital mutilated females and non-genital mutilated females, in personal subjects, marital relationship, solving problem, financial management, leisure, sexual relationship, and relatives and friends and also the total grades of marital satisfaction. So, genital mutilation was one of the basic factors in marital dissatisfaction. This result was similar to those of Alariqi, DeMaria (7, 14). They have presented that sexual satisfaction has a significant role in marital satisfaction. One of the marital satisfaction items was sexual satisfaction, and it was in the lowest level in genital mutilated females, the results of this research were similar to those of DeMaria who explained that there was a positive relation between high satisfaction in life and high satisfaction in sexual life (14). Also, the results of this research were similar to those of Alariqi in Yemen. She found that 24% of Yemeni females were circumcised or genitally mutilated and dissatisfaction of sexual relationship in them was more than females who did not mutilate (7). Also, Khodabakhshi Koolaee in their research “a report of Female Genital Mutilation in Kurdish females” concluded that the Kurdish females who experienced female genital mutilation reported problems in sexual satisfaction. They very hardly reached the orgasm and in general didn’t have orgasm regularly. In addition, they had problems in sexual function with their husbands (5). According to the results of Table 3, there was a meaningful difference between genital mutilated females and non-genital mutilated females, in physical symptoms and sleep disorders and also total grades of mental health. The results indicated that according to comparison of average scores of both groups, non-genital mutilated females had more mental health than the other groups. The results of research were consistent with those of Elsadavi (15). She showed that genital mutilation has the harmful effects over females’ physical and mental health and it may give sexual shocks in girls and it also decreases the female’s potential to reach an orgasm. She also found that female genital mutilation did not help to decrease of external genital organism’s cancer (15). Also according to UNICEF reports this action violates the female basic rights and it really hurts their health (6). Besides, Maslovskaya, Brown and Padmadas have shown that FGM increases the danger of suffering Human immunodeficiency virus (HIV). Their results indicated that there was a significant statistical relation between FGM and HIV. Also, they showed that possibility of suffering from HIV among young and old females who went through genital mutilation operation were a lot more than the females who did not (16). In general, it is worthy to point out that doing this inhuman action to women and young girls is an obvious sign of ignoring human rights and the right to have healthy and satisfying sexual relationship among women. Also, female genital mutilation not only affects the fulfillment of sexual function among women but also, influences their marital satisfactions. When people have not experienced the satisfaction in their marital life it may be the effect their mental health. Awareness of harmful consequences of this operation could be vital to prevent FGM among minority societies and the irrational believes which rooted in wrong customs. To implement the current study there were some problems and limitations. One of them was to complete the questionnaires. Some of the participants feared to tell the truth. As female Genital Mutilation is forbidden by government, the participants were feeling threatened. Also, the interviews women were time consuming, because some participants were uneducated and interviewers had to read all of items for them.
